# Come together: collaborative actions to bridge the gap between implementation research and public health practice

**DOI:** 10.3389/fpubh.2024.1424900

**Published:** 2024-06-13

**Authors:** Robert Larsson

**Affiliations:** Division of Public Health Sciences, School of Health, Care and Social Welfare, Mälardalen University, Västerås, Sweden

**Keywords:** collaboration, health equity, health promotion, implementation, practice, public health, research-practice gap

## Introduction

In October 2023, the third edition of the impactful book “Dissemination and Implementation Research in Health: Translating Science to Practice” was published (1). The book effectively points toward the advancements and knowledge gaps in the field of research. Knowledge development and support for implementation research and practice are desirable and necessary. Implementation science is a rapidly developing research field with immense potential to improve the implementation of public health policies, programs, and practices, thereby having an impact on population health. However, a key task that needs to be performed in public health and implementation science is to address and promote health equity (2, 3). With growing health inequities in many communities and societies, it is crucial to focus explicitly on health equity when implementing and putting public health actions into practice. In this context, it should also be underlined that public health practitioners are implementing and scaling “things” (i.e., public health actions) whether they are using implementation science or not, and the number of public health practitioners far outweighs the number of implementation researchers. Viewed positively, this means potential for improved population health impact and increased health equity if the implementation competence is strengthened among public health practitioners.

At the same time, there are worrying signs of a “second implementation gap,” and these signs are not explicitly addressed in the Brownson et al. (1) book. This gap refers to the *recreation* of the research-practice gap, a challenge that implementation research was originally intended for and should help to overcome. As implementation science has matured, various researchers have contributed to thoughtful self-reflection (4, 5), including those highlighting the “second implementation gap” (6–9). In this article, I continue this path and make a specific call to public health researchers and practitioners to be aware of the “second implementation gap” and suggest actions for handling the gap in public health.

## Implementation research and the research-practice gaps

Simply put, implementation means introducing new ideas into an organization, usually to try new methods, practices, technologies, systems, or policies (10). Implementation science involves research on processes and outcomes when research-based knowledge and evidence are introduced and used in different settings (11). Thus, implementation research aims to bridge the much-discussed “gap” between research and practice. Implementation is often demanding, and failure is common when introducing new methods and practices. For example, a decision to implement a new school-based method taken by upper-level school management involves teachers and school health professionals at lower organizational levels *doing* new “things,” but often without the resources to manage the new method. Given the risk of failing, implementation research is sometimes referred to as “misery research.”

It is common to say that it takes an average of 17 years for research to become routine in health care practice (12). While the time estimate is debatable, the implementation research goal is to help practitioners, managers, policymakers, and researchers anticipate common barriers and troubleshoot strategies for successful implementation. Finding solutions is crucial to shorten the time for uptake of research and evidence and for integration in practice and policy in public health and other health-related areas.

Recently, a “new” research-practice gap seems to have emerged in implementation research and practice despite commendable attempts to simplify the sometimes inaccessible and jargon-ridden implementation literature for practitioners (13). The research community discusses this problem as a “second implementation gap” or a “paradox” referring to a *recreation* of the research-practice gap (6–9). It is easy to see the irony in that research intended to bridge the research-practice gap has come to distance itself from the practice it is meant to improve. The fact that there currently are up to 200 theoretical models and frameworks to guide, analyze, and evaluate implementation in the health field effectively illustrates the problem. While some models and frameworks are used frequently, such as the Consolidated Framework for Implementation Research [CFIR, (14)], the Quality Implementation Framework [QIF, (15)], and the Reach-Effectiveness-Adoption-Implementation-Maintenance (RE-AIM) framework (16, 17), others have limited use (18). Also, few of these models and frameworks are known, used and fully relevant for public health practitioners (19). That said, it should be noted that theoretical and applied implementation researchers and public health practitioners are at different places on the translational spectrum (5). It means that researchers might find themselves on the basic science research to translation to practice and communities' spectrum, whereas public health practitioners usually are working on the translation to practice and community spectrum. Fortunately, implementation researchers have highlighted the problem of growing distance to the real-world practice where the research is to be used and have proposed both research agendas based on practitioners' perspectives (20) and ways for researchers and practitioners to come together.

## Collaborative actions

Inspired by Lyon and colleagues (7), I propose four bridging actions to increase collaboration between researchers and practitioners, with a focus on implementation in public health:

1. *Offer interprofessional learning opportunities:* Public health practitioners need better learning opportunities and working conditions to access new implementation knowledge. Many practitioners have extensive experience-based knowledge about implementation, but it is not common for them to be given time to read and familiarize themselves with research. Implementation science is a rapidly emerging knowledge field, and therefore it can be difficult for practitioners to keep up to date. Professional development can take place in diverse ways, such as courses, workshops, and webinars. Some areas to be included in professional development initiatives are (1) the use of existing theories, models, and frameworks (TMFs) for implementation, (2) teamwork, collaboration and communication between researchers and practitioners, and (3) how implementation research can become more relevant to practice (21). Previous research on implementation support highlights the value of looking beyond TMFs and including relationship-based competencies in professional development efforts (22). Professional development should also include critical perspectives on the collaboration between researchers, public health practitioners, and community members. Such perspectives are essential to avoid being over-optimistic or naïve about the collaboration. It is also valuable if different professions participate in professional development initiatives together, thereby creating opportunities for interprofessional learning between public health practitioners and other professions (e.g., psychologists, social workers, urban planners, and managers) involved when implementing public health actions. Preferably, such learning opportunities are organized by or involve implementation researchers who might also get insights and learn to improve the practical relevance of their research. It is also important to bring well-functioning learning initiatives to scale.
2. *Build collaborative teams of researchers and practitioners:* Implementation is a team sport. People with different knowledge, experiences, skills, and professional backgrounds need to work together for successful implementation. Collaboration applies to both practice and research, where it can be beneficial to work in interprofessional and interdisciplinary teams (7, 23, 24). Public health practitioners and researchers also need to form joint teams, collaborate to solve complex public health problems, and research implementation processes, outcomes, strategies, and challenges in practice. While some research and development environments and research teams work like this today, there may be room for improvement, for instance, in addressing research questions generated by practitioners (20). The collaboration between researchers and practitioners can contribute to mutual understanding, and involving implementation researchers could mean that they better understand public health practice. Previous studies on implementation support practitioners show that they prefer co-creation and exchange models in implementation (9, 22), which should also be valuable for public health practitioners.
3. *Prioritize pragmatism:* There is a need for research that utilizes pragmatic methods. Implementation research sometimes takes a pragmatic approach, but given the remaining gap between research and practice, more needs to be done. Pragmatic research in public health focuses on questions, data, and outcomes relevant to decision-making and action-taking (25). Two key words are “applicability” and “context.” Hence, pragmatic research aims to generate results relevant to policymakers, practitioners, and citizens but still generated with scientific rigor. For example, this may involve using short, simple, yet scientifically reliable questionnaires in the research. Pragmatic research is well-aligned with implementation research and practitioners should be included in the research process to achieve the above aims of practical relevance (see also point 1 and 2).
4. *Build communities of practice*: Many public health practitioners are engaged in implementation initiatives with varying levels of complexity. The idea is to create a “community of practice” (26) for knowledge sharing, experiences, and support on implementation in public health. Such communities can be formed globally, nationally, regionally, and locally using digital workspaces. Communities can help create new relationships between researchers and public health practitioners who in their daily work, are involved in implementation. Here it is worth mentioning that there are already existing networks and communities on implementation science and practice around the world. For example, hubs are connected to universities (e.g., Active Implementation Hub at the University of North Carolina, Chapel Hill in the USA, and the Health Implementation Research Hub at the University College Cork in Ireland) and there are also societies (e.g., Society for Implementation Research Collaboration and Global Implementation Society). So, an important task is to ensure that these networks and communities are known and open to public health practitioners and include public health initiatives.

## Discussion

Researchers and public health practitioners need to work together to bridge both the “first” and “second implementation gap” in public health. For this to happen, managerial and political support is needed. Ultimately, improved researcher-practitioner collaboration aims to ensure that evidence-based public health programs, practices, and policies are implemented as effectively as possible and can contribute to good and equitable health for the entire population. As we know, public health interventions work more effectively in more favored groups in society (e.g., high socioeconomic status groups), and effective public health implementation also risks increasing existing health inequities rather than reducing them. Therefore, improved knowledge and rapprochement between implementation research and public health practice are crucial to address health equity and societal challenges and to contribute to developing healthy and sustainable societies.

## Author contributions

RL: Conceptualization, Writing – original draft, Writing – review & editing.
